# Oblique lateral internal fusion combined with percutaneous pedicle screw fixation in severe lumbar spinal stenosis: clinical and radiographic outcome

**DOI:** 10.1186/s13018-023-04373-5

**Published:** 2023-11-20

**Authors:** Chen Liu, Yin Geng, Yifeng Li

**Affiliations:** 1 Department of Spine Surgery, First Affiliated Hospital of Wannan Medical College, No. 2 Zheshan West Road, Wuhu, 241001 Anhui China; 2 Spine Research Center of Wannan Medical College, No. 22 Wenchang West Road, Wuhu, 241001 Anhui China; 3 Key Laboratory of Non-Coding RNA Transformation Research of Anhui Higher Education Institution, Wannan Medical College, Wuhu, 241001 Anhui China

**Keywords:** OLIF, Indirect compression, Severe lumbar stenosis

## Abstract

**Background:**

Oblique lumbar interbody fusion (OLIF) has been a popular technique for treating lumbar degenerative diseases. Previous studies have shown its efficiency in lumbar spinal stenosis; yet, only a few studies have investigated its application to severe lumbar spinal stenosis. Herein, we investigated the clinical and radiographic outcome of OLIF with percutaneous pedicle screws in the treatment of severe lumbar spinal stenosis.

**Methods:**

A total of 15 patients who underwent OLIF with percutaneous pedicle screws were retrospectively analysed. All patients were diagnosed with severe lumbar stenosis (Schizas grade C or D) through preoperative magnetic resonance image (MRI) and received OLIF combined with percutaneous pedicle screw surgery. Clinical outcomes, including visual analogue scale (VAS)-back and VAS-leg scores, and Oswestry Disability Index (ODI), as well as mean disc height (DH), mean foraminal height (FH), segmental lumbar lordosis (SLL) and cross-sectional area (CSA) of the spinal canal, were analysed before and after surgery and at the last follow-up. Intraoperative data, complications and fusion rate were also investigated.

**Results:**

OLIF combined with percutaneous pedicle screws was performed on 18 segments in 15 patients. Mean follow-up was 23.1 ± 4.6 months (range 15–29 months). VAS-back, VAS-leg, and ODI scores were significantly improved at the last follow-up. DH increased from 8.86 ± 3.06 mm before surgery to 13.31 ± 2.14 mm after; at the last follow-up, DH was 11.69 ± 1.87 mm. FH increased from 17.85 ± 2.26 mm before surgery to 22.09 ± 1.36 mm after; at the last follow-up, FH was 20.41 ± 0.99 mm. CSA of the spinal canal increased from 30.83 ± 21.15 mm^2^ before surgery to 74.99 ± 33.65 mm^2^ after the operation and 81.22 ± 35.53 mm^2^ at the last follow-up. The segmental LL before surgery, after surgery and at last follow-up was 20.27 ± 6.25 degrees, 20.83 ± 6.52 degrees and 19.75 ± 5.87 degrees, respectively. All patients have gained fusion at the last follow-up.

**Conclusion:**

OLIF with percutaneous pedicle screws could achieve satisfactory clinical and radiographic effects through indirect compression by increasing DH, FH and CSA of the spinal canal in severe lumbar stenosis patients.

## Introduction

Lumbar stenosis refers to a back condition that commonly affects the legs and occurs due to the narrowing of the area that contains the nerves or spinal cord. Symptoms of lumbar stenosis, such as low back pain and intermittent neurogenic claudication, result from compression of nerves and blood vessels in the spinal canal and neural foramen, which often occur secondary to ligament hypertrophy or lumbar spondylolisthesis. Severe stenosis can cause significant discomfort, decrease daily living activities and lead to poor quality of life [1–4]. The main treatment methods for lumbar spinal stenosis include conservative and surgical approaches. The conservative treatment mainly improves the symptoms through absolute bed rest, the functional exercise of lumbar dorsal muscles, etc., while the surgical approach is the most effective way to relieve the neurological symptoms and the compression of the lumbar spinal canal for patients whose neurological symptoms have not significantly improved or even worsened [5]. Posterior lumbar interbody fusion (PLIF) with bilateral facetectomy and transforaminal lumbar interbody fusion (TLIF) with unilateral facetectomy are regarded as widely used orthopaedic spine surgeries for lumbar stenosis, which can obtain satisfactory and certain clinical effects [6]. However, this approach requires paravertebral muscle detachment, nerve root retraction, and manipulation of the dura [7], which may lead to major trauma and potential nerve root and dural sac injury. Direct lateral interbody fusion/extreme lateral interbody fusion (DLIF/XLIF) is a minimally invasive approach for treating lumbar stenosis that has achieved excellent clinical and radiographic results [8–10]. DLIF/XLIF differs from traditional procedures since the surgeon accesses the space between each spinal disc from the patient's side, sparing major back muscles, bones, and ligaments. However, this approach has been associated with a relatively high incidence of psoas major and lumbar plexus injury, even when neuromonitoring is utilized [11].

In 2012, Sliver et al. [12] reported the efficacy of oblique lumbar interbody fusion (OLIF) in treating lumbar degenerative diseases. The major advantages of OLIF are less trauma, shorter operation time, less intraoperative bleeding, shorter hospital stay, and faster postoperative recovery. So far, several studies have reported certain beneficial effects of applying OLIF in lumbar spinal stenosis [13–16]. However, due to the characteristics of indirect decompression, whether OLIF could achieve good clinical efficacy in treating patients with severe lumbar spinal stenosis remains debatable. Heo et al. [17, 18] suggested that OLIF was unsuitable for patients with severe canal stenosis. On the contrary, Woo et al. [19] advocated that indirect decompression principles might be applied in patients with severe spinal canal stenosis, even Schizas grade D. Besides, another study indicated that the efficiency of OLIF for severe central stenosis was favourable [20].

This study investigated the clinical efficacy and radiographic results of OLIF combined with percutaneous posterior pedicle screws in treating severe lumbar spinal stenosis.

## Methods

### Patients

A total of 15 patients with severe lumbar spinal stenosis diagnosed using MRI who underwent OLIF combined percutaneous posterior pedicle screw surgery in Yijishan Hospital of Wannan Medical College from November 2020 to November 2021 were retrospectively analyzed. Inclusion criteria were: (1) preoperative MRI indicated severe lumbar spinal stenosis (Schizas grade C or D) [21]. Patients were diagnosed with lumbar spinal stenosis or lumbar spinal stenosis secondary to lumbar spondylolisthesis; (2) Neurological symptoms were still obvious or even aggravated after receiving conservative treatment; 3) patients who have been followed up for at least 1 year and had complete follow-up data. Exclusion criteria were: (1) patients with a bone fusion of facet joint from the preoperative computed tomography (CT) images; (2) intervertebral disc was severely calcified; (3) with lumbar traumatic injury, tumour, or infection.

### Operation procedure

**Fig. 1:**
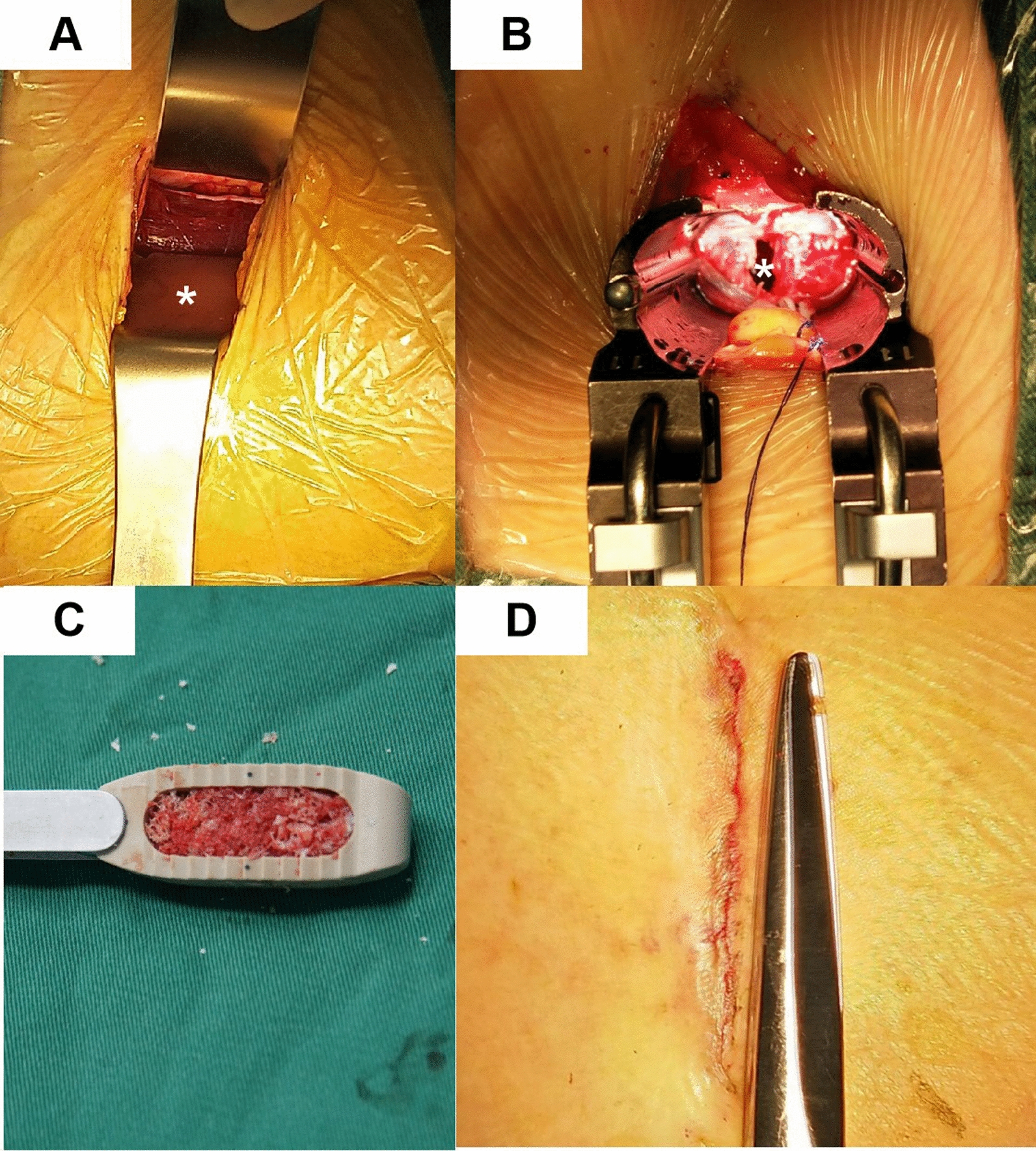
OLIF surgery procedure. **A** The psoas muscle was exposed (*, psoas major muscle). **B** The operative space was dilated with the retractors (*, intervertebral disc space). **C** The huge OLIF cage is filled with allografts. **D** The postoperative incision

OLIF was performed under general anaesthesia with the patient in the right decubitus position. A 4-cm transverse skin incision was made in the horizontal plane overlying the involved segment or segments on the left lateral abdomen. After blunt dissection of the abdominal wall muscles, the psoas major was retracted posteriorly, and the abdominal vessels were retracted anteriorly. Sequential dilators and retractors were placed over a guide needle after it was inserted in the middle of the target intervertebral disc space under fluoroscopic guidance. The disc and cartilaginous endplates were removed to expose the bony endplates. A wide lordotic Clydesdale® intervertebral fusion cage (Medtronic, Memphis, Tennessee) was packed with allograft bone and inserted into the target disc space under fluoroscopic guidance. The wound was closed in layers (Fig. 1). No intraoperative neuromonitoring was performed during the OLIF procedure. Next, the patient was placed to the prone position, and a bilateral percutaneous pedicle screw system was performed. The patients were encouraged to do out-of-bed activity the day after the operation with the help of waist protection, and they were not allowed to bend and bear weight 3 months after the operation.

### General characteristics and perioperative data collection

The general baseline demographics, including age, sex and BMI, were investigated. Perioperative data included operative level, estimated blood loss, and intra- and postoperative complications (such as new or aggravated nerve damage, incisional infection, hematoma, vascular injury, ureteral injury, cage displacement and subsidence, and vertebral fracture). The degree of cage subsidence was determined according to Marchi’s study: Grade 0 (0–24%), grade I (25–49%), grade II (50–74%), and grade III (75–100%) [22].

### Clinical and radiographic outcomes

Clinical outcomes were evaluated using 10-point back and leg visual analogue scale (VAS) scores and the Oswestry Disability Index (ODI). Scores were obtained before surgery and at the last follow-up. Standing anterior–posterior and lateral lumbar radiography was performed before surgery, 1 day and 3 months after surgery and at the last follow-up. The MRI was performed before and 1 day after surgery. The lumbar spine CT was performed before and 1 day after surgery and at the last follow-up. The CT performed at the last follow-up visit was used to assess bony fusion. Mean disc height (DH; mean value of the leading and trailing edge height of the intervertebral disc), foraminal height (FH; the distance from the lower position of the pedicle of the upper vertebra and the upper position of the pedicle of the lower vertebra), segmental lumbar lordosis (SLL), and cross-sectional area (CSA) of the spinal canal were recorded before and after surgery and at the last follow-up visit. All measurements were performed by independent radiologists using a PACS system, following a previous study protocol [23].

### Statistical analysis

Statistical analyses were performed using SPSS software version 19.0 (IBM Corp, Armonk, NY, USA). Measurements are expressed as means with standard deviation. Preoperative and last follow-up VAS scores and ODI were compared using the *t* test; DH, FH, SLL and CSA of the spinal canal were compared using analysis of variance. *P* < 0.05 represented statistical significance.

## Results

### Patient characteristics and surgical data

A total of 15 patients were included for analysis (7 women and 8 men; mean age of 62.8 ± 9.3 years). A total of 18 lumbar levels were treated (12 single-level and 3 double-level operations). The mean follow-up was 23.1 ± 4.6 months; the mean estimated blood loss was 76.7 ± 25.8 mL; and the mean length of hospitalization after the operation was 4.7 ± 1.2 days. Patient characteristics and surgical data are shown in Table 1.

**Table 1:** Patient demographic and treatment information

Characteristic	Statistic (*n* = 15)
Mean age (range)	62.8 ± 9.3
Female (%)	7 (46.7%)
BMI	23.5 ± 2.8
Levels per operation	
One level (% of cases)	12 (69.8)
Two levels (% of cases)	3 (30.2)
Operation levels	
L3/4 (% of levels)	3
L4/5 (% of levels)	15
Cage size	
8*45 mm	1
10*50 mm	1
12*50 mm	6
12*55 mm	2
14*50 mm	3
14*55 mm	5
Schizas classification	
C	7
D	8
Blood loss (range)	76.7 ± 25.8 (50–100) (mL)
Hospitalization after operation	4.7 ± 1.2 (days)
Follow-up	23.1 ± 4.6 ( months)

### Clinical and radiographic outcomes

VAS-back and VAS-leg scores and ODI were significantly lower at the last follow-up than before surgery (VAS-back score: 1.3 vs. 4.7; VAS-leg score: 1.4 vs. 4.8; *P* < 0.01; ODI: 12.8 vs. 34.1) (Table 2**)**. DH increased from 8.86 ± 3.06 mm before surgery to 13.31 ± 2.14 mm after; at the last follow-up, DH was 11.69 ± 1.87 mm. FH increased from 17.85 ± 2.26 mm before surgery to 22.09 ± 1.36 mm after; at the last follow-up, FH was 20.41 ± 0.99 mm. Both DH and FH were significantly higher after surgery; however, both decreased slightly between the day after surgery and the last follow-up, likely because of cage subsidence. The segmental LL before surgery, after surgery and at last follow-up was 20.27 ± 6.25 degrees, 20.83 ± 6.52 degrees and 19.75 ± 5.87 degrees, respectively; there were no significant differences among these results (all *P* > 0.05).

**Table 2:** VAS score and ODI index

	Before operation	Last follow-up	*P* value
VAS-back	4.7 ± 0.8	1.3 ± 0.5	0.000
VAS-leg	4.8 ± 1.0	1.4 ± 0.5	0.000
ODI	34.1% ± 3.2%	12.8% ± 1.8%	0.000

**Fig. 2:**
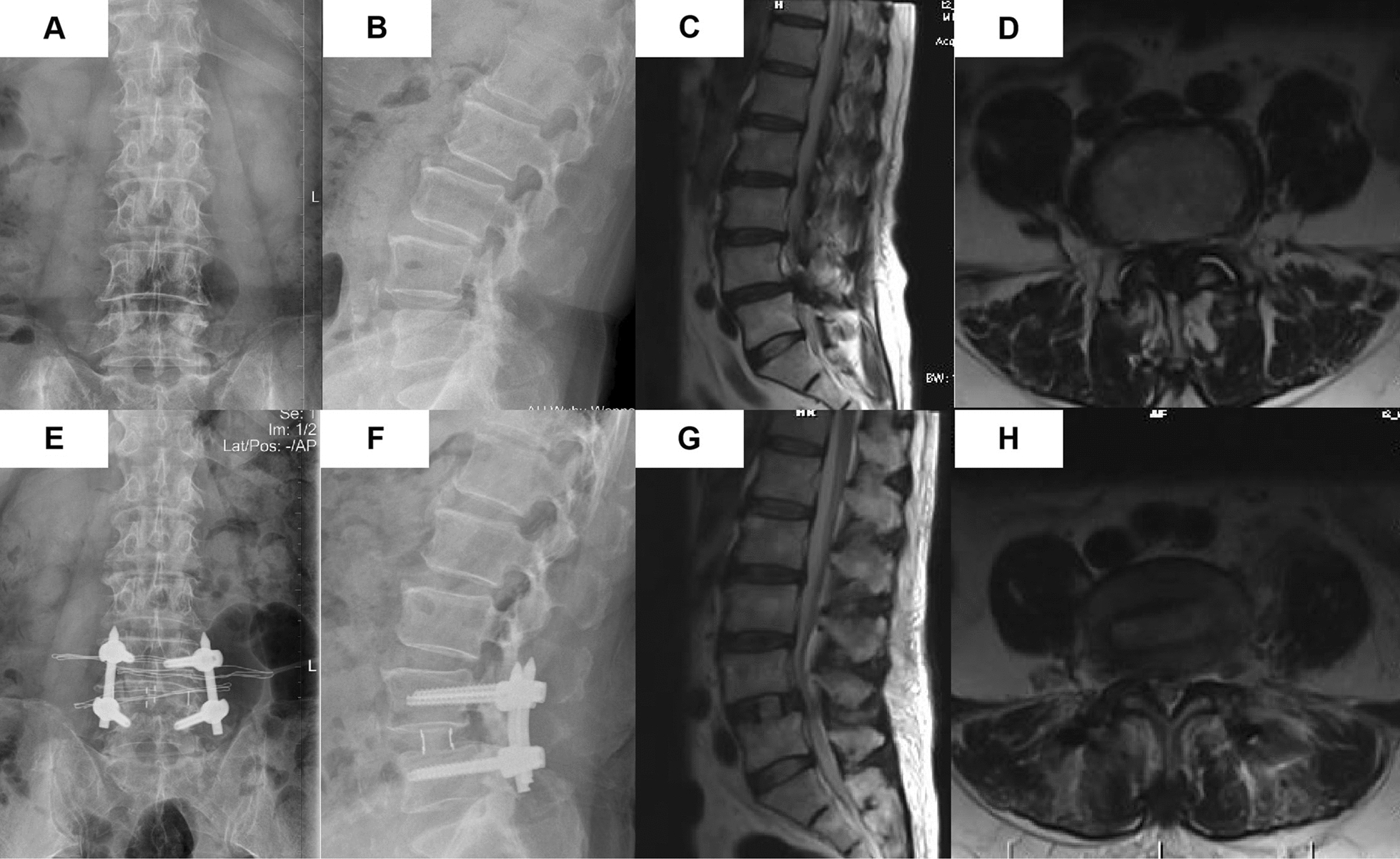
A typical case of OLIF. **A, B** The preoperative anteroposterior and lateral radiographs demonstrate Grade 1 spondylolisthesis at L4/5. **E, F** The preoperative sagittal and coronal MRI shows severe central stenosis (Schizas Grade D) at L4/5. **C, D** The postoperative anteroposterior and lateral radiographs one day after OLIF. **G, H** The preoperative sagittal and coronal MRI showed (Schizas Grade B) at L4/5

CSA of the spinal canal increased from 30.83 ± 21.15 mm^2^ before surgery to 74.99 ± 33.65 mm^2^ after the operation and 81.22 ± 35.53 mm^2^ at the last follow-up (Table 3). At the last follow-up, fusion was demonstrated on CT in 15 patients (100%). The imaging examination of a typical case is presented in Fig. 2. There were 7 Schizas grade C patients and 8 Schizas grade D patients before surgery. However, the spinal stenosis was preoperative Schizas grade A 6 and Schizas grade B 9, which is shown in Table 4.

**Table 3:** Radiographical results

	Before operation	After operation	Last follow-up	*P* value^*^	*P* value^**^	*P* value^***^
DH (mm)	8.86 ± 3.06	13.31 ± 2.14	11.69 ± 1.87	0.000	0.001	0.05
FH (mm)	17.85 ± 2.26	22.09 ± 1.36	20.41 ± 0.99	0.000	0.000	0.003
SLL (degrees)	20.27 ± 6.25	20.83 ± 6.52	19.75 ± 5.87	0.807	0.820	0.637
CSA (mm^2^)	30.83 ± 21.15	74.99 ± 33.65	81.22 ± 35.53	0.000	0.000	0.546

*P* value^*^ stands for the result between the values before operation and after operation

*P* value^**^ stands for the result between the values after operation and last follow-up

*P* value^***^ stands for the result between the values before operation and last follow-up

**Table 4:** Spinal stenosis results

	Before operation	After operation
Schizas grade A	0	6
Schizas grade B	0	9
Schizas grade C	7	0
Schizas grade D	8	0

### Complications

Intraoperative complications occurred in one patient (6.7%). The patient experienced endplate injury and was treated using a thoracolumbar brace. Postoperative complications such as wound infection, retrograde ejaculation, and psoas major hematoma were not observed.

Left sympathetic chain injury occurred in one patient (6.7%); the patient recovered within one week. One patient (6.7%) reported left thigh pain after surgery; the patient recovered within 5 days. Also, cage subsidence occurred in two patients (13.3%); subsidence was < 24% of the cage height in both cases. No patients with subsidence experienced a recurrence of symptoms during the follow-up (Table 5).

**Table 5:** Intraoperative and postoperative complications

Complications	Intraoperative cases (%)	Postoperative cases (%)
End plate damaged	1 (6.7)	–
Left sympathetic nerve injury	–	1 (6.7)
Pain in front of left thigh	–	1 (6.7)
Cage subsidence	–	2 (13.3)

## Discussion

The efficacy of OLIF in treating lumbar degenerative diseases has been recognized by many scholars [24, 25]. Hiyama et al*.* [26] assessed 80 patients into severe stenosis, moderate stenosis, and mild stenosis groups; the cluster analysis results indicated that lateral lumbar interbody fusion (LLIF), a direct decompression method, could achieve similar results in terms of central canal area, canal diameter, disc angle and anterior, posterior, and average disc heights when treating all three groups. Yet, so far, only a few studies have investigated the treatment effect of OLIF for severe lumbar spinal stenosis. In our previous study, we found that stand-alone OLIF was an effective and safe option for treating adult degenerative scoliosis in carefully selected patients [27]. In this study, we further explored the effect of OLIF indirect decompression with percutaneous posterior pedicle screws by comparing clinical data, including VAS-back, VAS-leg and ODI and preoperative, postoperative, and last follow-up DH, FH and CSA of the spinal canal in this study. We observed that the clinical effect of indirect decompression one year after stand-alone OLIF operation was satisfactory.

As the X-ray of a typical case (Fig. 2**)** showed, DH and FH were significantly higher immediately after the operation, which is attributed to the huge OLIF cage placement. Besides, the MRI image after the operation demonstrated that the CSA of the spinal canal was expanded due to the stretching and thinning of the ligamentum flavum. The increased FH and CSA of the spinal canal could relieve lower limb pain symptoms caused by intervertebral foramen and central canal stenosis. Therefore, we concluded that OLIF could achieve indirect decompression by increasing DH and FH and restoring ligamentum flavum length and tension to increase the CSA of the spinal canal. Similar results were reported by previous studies. Sato et al*.* [28] evaluated 20 lumbar degenerated spondylolisthesis patients who underwent OLIF combined with percutaneous pedicle screw fixation, and the disc height and spinal canal area were improved after surgery. In Fujibayashi’s study, 28 patients received oblique lateral interbody fusion combined with percutaneous pedicle screw fixation, and the results showed that the mean CSA increased from 99.6 mm preoperatively to 134.3 mm postoperatively [10]. In a study that included 25 patients who underwent OLIF with and without posterior internal fixation, Lin et al*.* [29] reported 4 mm increases in mean DH and FH after surgery. In addition, Wang et al*.* [30] reported an increase in DH and CSA of the spinal canal in 13 patients who underwent stand-alone OLIF for treatment of adjacent segment disease. The above studies have shown that even stand-alone OLIF without posterior pedicle screws could achieve satisfactory clinical effects for lumbar stenosis patients.

The last follow-up showed a decrease in DH and FH; the average DH was 11.69 mm, similar to the average height of the intraoperative fusion cage (12.56 mm). The results revealed no obvious fusion cage subsidence. On the contrary, there was an improvement in CSA of the spinal canal at the last follow-up, which may be due to improved VAS-back, VAS-leg scores and ODI at the last follow-up. We could infer that although the height of the intervertebral disc decreased during the follow-up, the volume of the vertebral canal further increased due to the remodelling of the ligamentum flavum. Similar results were reported in Mahatthanatrakul’s study [31]. The mean DH of 17 patients who received OLIF surgery increased from 7.6 ± 1.6 to 11.6 ± 1.7 mm immediately after the operation but decreased to 10.1 ± 1.6 mm during the follow-up. Yet, the CSA increased from 96.9 ± 54.9 to 136.0 ± 72.7 mm^2^ immediately after an operation and 171.4 ± 76.10 mm^2^ at the last follow-up. Furthermore, Limthongkul et al*.* [32] explored thirty-five patients who underwent extreme lateral interbody fusion or OLIF with percutaneous pedicle screw fixation and found that a mean CSA of thecal sac increased from 93.1 ± 43.0 mm^2^ to 127.3 ± 52.5 mm^2^.

Our study used a 6° lordotic cage, but the results indicated no significant improvement in SLL after OLIF surgery and at the last follow-up. We infer that we implanted the huge OLIF cage behind the intervertebral space as much as possible during the surgery, which is not conducive to the recovery of lumbar lordosis. In addition, previous literature reported that LLIF had great capacity in correcting coronal deformity and limited ability to improve sagittal correction [33].

Significant osteophyte ossification of the posterior longitudinal ligament has been regarded as a risk factor for indirect decompression failure. However, in this study, patients with severe lumbar spinal stenosis did not have ossification of the posterior longitudinal ligament. On the contrary, the patient's symptoms were attributed to hypertrophy of ligamentum flavum or stenosis of the intervertebral foramen. The immediate enlargement of CSA of the spinal canal after the operation may be due to the increase in intervertebral height, which leads to debuckling of the ligamentum flavum and posterior longitudinal ligaments. Woo et al*.* [19] reviewed 62 patients who underwent L4/5 OLIF, including 24 grade 3 patients and 16 grade 4 patients before surgery, and the results showed that only 2 patients were grade 3 and no patient was grade 4. Besides, Shimizu et al*.* [34] found that clinical symptomatology would be resolved even if the expansion was subtle, thus suggesting that the degree of symptom improvement in patients with lumbar spinal stenosis is not positively correlated with the area of the enlarged spinal canal. For example, Lin et al*.* [29] compared OLIF and minimally invasive transforaminal lumbar interbody fusion (MIS-TLIF) in the treatment of lumbar stenosis and found that OLIF may achieve equivalent clinical and radiologic outcomes compared with MIS-TLIF when the stenosis is minimal. In addition, Shimizu et al*.* [20] made the comparison between OLIF and TLIF/PLIF in the treatment of patients with severe central canal stenosis and concluded that OLIF has a comparable short-term clinical outcomes in the treatment of severe degenerative lumbar stenosis compared with conventional TLIF and/or PLIF. Surprisingly, the radiographic outcomes in this study were even better in the OLIF group than in the TLIF/PLIF group. Similar results were reported by Hiyama et al*.* [35]*,* who proved that XLIF with indirect decompression may lead to lower blood loss and low back pain compared with minimally invasive surgery MIS-TLIF in patients with degenerative spondylolisthesis. Moreover, Gagliardi et al*.* [36] conducted a systematic review of the literature on studies assessing patients diagnosed with degenerative lumbar spinal stenosis and instability and treated with indirect or direct decompression and fusion surgery and found that indirect decompression surgery could achieve similar clinical efficiency with lower intraoperative blood loss and less surgical time compared with direct decompression surgery. Goel et al*.* [37] invocated that spinal instability was the nodal point of the pathogenesis of spinal degeneration-related lumbar canal stenosis and suggested that lumbar stenosis can be treated via fixation of the involved spinal segments. The scholar suggested that clinical score improvement may be more associated with the effect of stabilization than that of neural decompression. We believe that stability and indirect decompression have equally important roles in treating severe lumbar spinal stenosis, and we speculate that there is a critical value for the symptoms caused by severe spinal stenosis. A good clinical efficacy can be achieved if the spinal canal volume exceeds this critical value after indirect decompression. Shimizu et al*.* [34] showed that a small preoperative CSA tends to have greater expansion, implying that a small preoperative CSA is not necessarily a contraindication for indirect decompression. Besides, we found that the CSA of the spinal canal is not only improved immediately after surgery but also further expanded in the last follow-up. Yet, at present, there is no clear explanation for ligamentum flavum remodelling. Based on the finding that the fusion of non-ossified segment of OPLL could decrease OPLL thickness [38], some scholars suggested a hypothesis that the spinal fusion may stop the progression of ligamentum flavum hypertrophy by reducing mechanical stress to the ligamentum flavum [31, 39]. Therefore, supplemental screw fixation should be strongly recommended for patients with severe lumbar stenosis. Reliable fixation can prevent cage subsidence but also remodel the ligamentum flavum, with the ultimate goal of maintaining or even expanding the CSA of the spinal canal to alleviate symptoms of severe lumbar stenosis patients. In fact, previous studies discussed the ideal cage position in lateral lumbar interbody fusion. For example, Park et al*.* [40], Otsuki et al*.* [41], and Qiao et al*.* [42] suggested placing the cage in the anterior 1/3 of disc space to achieve segmental lordosis in LLIF surgery in their studies. However, Hiyama et al*.* [43] suggested that putting the cage in the posterior position in LLIF surgery might be effective for expanding the CSA. In this study, we suggested placing the cage as posteriorly as possible in lumbar stenosis patients to achieve maximum disc space distraction. In order to avoid the injury of the ipsilateral and contralateral nerves, the fusion cage should be vertically implanted into the intervertebral space.

Complications have always been an important concern for clinical researchers. OLIF complications can be classified as approach- or cage-related. The incidence of approach-related complications in our study was 13.3%, including left sympathetic nerve injury in one patient (6.7%) and pain in front of the left thigh in another patient. Similarly, Abe et al*.* [44] reported a 16.1% incidence of approach-related complications in a study of 155 OLIF patients. Cage-related complications were most common. The cage subsidence resulting in disc height loss and vertebral body fracture is the major complication of LLIF, which may lead to failure of clinical efficacy. Wu et al*.* [45] performed a systematic review to investigate risk factors of cage subsidence. They concluded that female patients, patients with poor bone density, and patients older than 65 have a higher risk of developing complications. In the present study, the incidence rate of cage subsidence was 13.3%. Ahmadian et al*.* [46] evaluated the clinical outcomes of patients who received stand-alone lateral interbody fusion, and the results indicated that 30% of the patients had grade I and grade II subsidence. Zhang et al*.* [27] investigated the efficiency of stand-alone OLIF for the treatment of ADS and showed 16.7% cage subsidence. Cai et al*.* [47] explored the biomechanical differences among stand-alone OLIF, OLIF with lateral plate fixation, OLIF with unilateral pedicle screws fixation, OLIF with bilateral pedicle screws fixation and OLIF with translaminar facet joint fixation combined unilateral pedicle screws fixation in a three-dimensional nonlinear finite element model, finding that OLIF with bilateral pedicle screws fixation had the best ability in restoring lumbar stability and resisting cage subsidence. Previous studies also had similar reports. Zeng et al*.* [48] compared stand-alone OLIF and OLIF combined with posterior pedicle screw fixation and reported early complication incidence rates of 36.26% and 29.86%, respectively; the reason for the difference was a higher incidence of cage subsidence in the stand-alone OLIF group. In another study, Wang et al*.* [49] compared the efficacy of OLIF stand-alone with OLIF combined with percutaneous pedicle screw fixation (PPSF) in the treatment of discogenic low back pain. The cage subsidence was 28% in the OLIF stand-alone group, whereas the value decreased to 11.76% in the OLIF + PPSF group.

The ultimate goal of the operation was fusion, regardless of various kinds of fusion methods. The fusion rate was 100% at the last follow-up. Kim et al*.* [50] reported a fusion rate of 92.9% in a 12-month follow-up in 29 patients who underwent OLIF combined posterior fixation. Furthermore, Wang et al*.* [39] reported that the fusion rate of OLIF combined with PPSF was 94.12% at 12 months post-surgery. The value increased to 100.0% at the last follow-up. On the contrary, Roh et al*.* [51] reported that the radiological fusion rate was 77.1%, 91.4% and 94.3%, respectively, at the 1-year, 5-year and 10-year follow-up after investigating thirty patients who underwent minimally invasive transforaminal lumbar interbody fusion. Another study directly showed that the fusion rate of the OLIF group (87.2%) was significantly higher than the TPLIF group (57.4%) at one-year follow-up by comparing the clinical outcome of OLIF versus TPLIF in the treatment of severe lumbar stenosis [20]. Thus, we concluded that the larger OLIF cage could provide a more effective biological substrate for fusion. Similarly, Yuan et al*.* [52] found a 5-mm cage subsidence for every 1 mm^2^ increment of cage area needed an additional 8 N of force.

This study has a few limitations. First, this was a retrospective study with a small sample size. Second, the follow-up was short, with a mean follow-up time of 23.1 months. Third, the conventional PLIF/TLIF with direct decompression as a control group may be needed in the future studies to further confirm these findings. In the future study, we plan to compare the radiographic and clinical outcomes between OLIF combined with percutaneous pedicle screws with indirect compression and PLIF/TLIF with direct compression using a long-term follow-up to treat severe lumbar stenosis.

## Conclusion

Through indirect decompression, OLIF combined with percutaneous posterior pedicle screws could significantly improve DH, FH, CSA, VAS and ODI scores. Our data suggest that this technique is safe, useful, and minimally invasive for treating severe lumbar stenosis.

## Data Availability

The datasets used in the current study are available from the corresponding author.

## Abbreviations

OLIF: Oblique lumbar interbody fusion
VAS: Visual Analogue Scale
ODI: Oswestry Disability Index
DH: Disc height
FH: Foraminal height
SLL: Segmental lumbar lordosis
CSA: Cross-sectional area
PLIF: Posterior lumbar interbody fusion
TLIF: Transforaminal lumbar interbody fusion
ALIF: Anterior lumbar interbody fusion
LLIF: Lateral lumbar interbody fusion
