# Correction: Oblique lateral internal fusion combined with percutaneous pedicle screw fixation in severe lumbar spinal stenosis: clinical and radiographic outcome

**DOI:** 10.1186/s13018-023-04414-z

**Published:** 2023-12-11

**Authors:** Chen Liu, Yin Geng, Yifeng Li

**Affiliations:** 1 Department of Spine Surgery, First Affiliated Hospital of Wannan Medical College, No. 2 Zheshan West Road, Wuhu, 241001 Anhui China; 2 Spine Research Center of Wannan Medical College, No. 22 Wenchang West Road, Wuhu, 241001 Anhui China; 3 Key Laboratory of Non-Coding RNA Transformation Research of Anhui Higher Education Institution, Wannan Medical College, Wuhu, 241001 Anhui China

**Correction: Journal of Orthopaedic Surgery and Research (2023) 18:882** 10.1186/s13018-023-04373-5

Following publication of the original article [1], the authors identified an error in Fig. 1. The correct figure is given below.

**Fig. 1:**
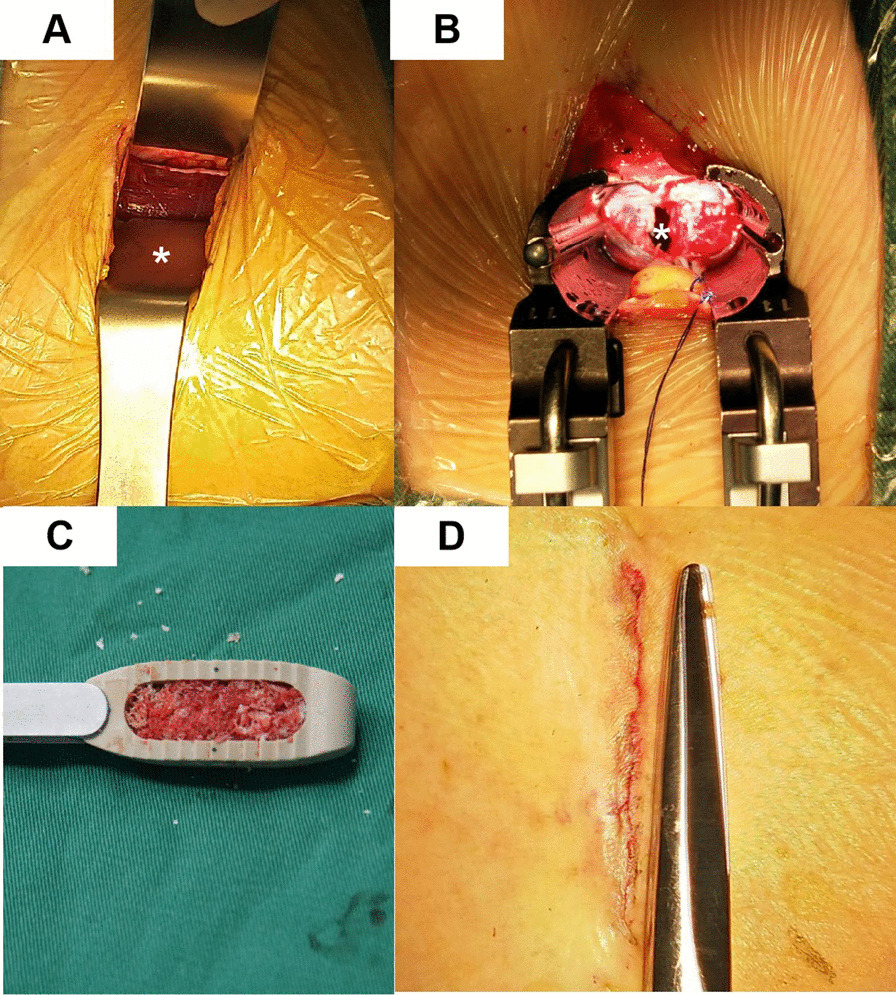
OLIF surgery procedure. **A** The psoas muscle was exposed (*, psoas major muscle). **B** The operative space was dilated with the retractors (*,intervertebral disc space). **C** The huge OLIF cage is flled with allografts. **D** The postoperative incision

The original article [1] has been revised.

