# Correction: Oblique lateral internal fusion combined with percutaneous pedicle screw fixation in severe lumbar spinal stenosis: clinical and radiographic outcome

**DOI:** 10.1186/s13018-024-05276-9

**Published:** 2024-11-24

**Authors:** Chen Liu, Yin Geng, Yifeng Li

**Affiliations:** 1 Department of Spine Surgery, First Affiliated Hospital of Wannan Medical College, No. 2 Zheshan West Road, Wuhu, 241001 Anhui China; 2 Spine Research Center of Wannan Medical College, No. 22 Wenchang West Road, Wuhu, 241001 Anhui China; 3 Key Laboratory of Non-Coding RNA Transformation Research of Anhui Higher Education Institution, Wannan Medical College, Wuhu, 241001 Anhui China

**Correction: Journal of Orthopaedic Surgery and Research (2023) 18:882** **https://doi.org/10.1186/s13018-023–04373-5**

Following publication of the original article, the authors identified an error in the fifth line in Methods Patients part.

1. Inclusion time for the patient was November 2020 to November 2021, but it was mistakenly indicated it as November 2020 to June 2021. (The Fifth line in Methods Patients part)
2. The patient’s age was 62.8±9.3 instead of 63.8±8.9. (The first line of Table 1 and the second line in Results Patient characteristics and surgical data part). These adjustments will not affect the results or conclusions of the article.

**Table 1:** Patient demographic and treatment information

Characteristic	Statistic (n = 15)
Mean age (range)	62.8±9.3
Female (%)	7 (46.7%)
BMI	23.5±2.8
Levels per operation	
One level (% of cases)	12 (69.8)
Two levels (% of cases)	3 (30.2)
Operation levels	
L3/4 (% of levels)	3
L4/5 (% of levels)	15
Cage size	
8 * 45 mm	1
10 * 50 mm	1
12 * 50 mm	6
12 * 55 mm	2
14 * 50 mm	3
14 * 55 mm	5
Schizas classification	
C	7
D	8
Blood loss (range)	76.7±25.8
Hospitalization after operation 4.7±1.2 (days)	(50–100) (mL)
Follow-up	23.1±4.6 ( months)

**Table 2:** VAS score and ODI index

	Before operation	Last follow-up	P value
VAS-back	4.7 ± 0.8	1.3 0.5	0.000
VAS-leg	4.8 ± 1.0	1.4 ± 0.5	0.000
ODI	34.1% ± 3.2%	12.8% ± 1.8%	0.000

**Table 3:** Radiographical results

	Before operation	After operation	Last follow-up	P value^*^	P value^**^	P value^***^
DH (mm)	8.86 ± 3.06	13.31 ± 2.14	11.69 ± 1.87	0.000	0.001	0.05
FH (mm)	17.85 ± 2.26	22.09 ± 1.36	20.41 ± 0.99	0.000	0.000	0.003
SLL (degrees)	20.27 ± 6.25	20.83 ± 6.52	19.75 ± 5.87	0.807	0.820	0.637
CSA (mm^2^)	30.83 ± 21.15	74.99 ± 33.65	81.22 ± 35.53	0.000	0.000	0.546

P value^*^ stands for the result between the values before operation and after operation;

P value^**^ stands for the result between the values after operation and last follow-up;

P value^***^ stands for the result between the values before operation and last follow-up.

**Table 4:** Spinal stenosis results

	Before operation	After operation
Schizas grade A	0	6
Schizas grade B	0	9
Schizas grade C	7	0
Schizas grade D	8	0

**Table 5:** Intraoperative and postoperative complications

Complications	Intraoperative cases (%)	Postoperative cases (%)
End plate damaged	1 (6.7)	–
Left sympathetic nerve injury	–	1 (6.7)
Pain in front of left thigh	–	1 (6.7)
Cage subsidence	–	2 (13.3)

The original article has been corrected.

